# Loss to follow-up associated factors in patients with chronic pulmonary aspergillosis and its impact on the disease prognosis

**DOI:** 10.3389/fpubh.2022.1026855

**Published:** 2022-12-13

**Authors:** Shaoqiang Li, Ya Li, Zhengtu Li, Xin Yang, Yangqing Zhan, Weilong Li, Ye Lin, Feng Ye

**Affiliations:** ^1^State Key Laboratory of Respiratory Disease, National Clinical Research Center for Respiratory Disease, Guangzhou Institute of Respiratory Health, The First Affiliated Hospital of Guangzhou Medical University, Guangzhou Medical University, Guangzhou, China; ^2^General Practice Department, The First Affiliated Hospital of Guangzhou Medical University, Guangzhou, China

**Keywords:** chronic pulmonary aspergillosis, loss to follow-up, risk factor, prognosis, case fatality rate (CFR)

## Abstract

**Objective:**

Pulmonary aspergillosis is a rare but challenging pulmonary disease. The conditions of patients with chronic pulmonary aspergillosis (CPA) can be even more complicated. The mortality rate of CPA remains high, and the prognostic factors are not well established due to a high proportion of loss to follow-up. In this study, we aim to explore factors associated with loss to follow-up in CPA patients and their impact on the disease prognosis after withdrawing anti-fungal treatments.

**Methods:**

Patients with confirmed CPA, who were admitted to the Department of Respiratory and Critical Care Medicine, the First Affiliated Hospital of Guangzhou Medical University from March 2017 to November 2019, were enrolled in this prospective study. The enrolled patients were followed up for 6 months after discharge. For loss to follow-up patients, the reasons for loss to follow-up and their prognosis after withdrawing anti-fungal treatments during loss to follow-up were recorded by telephone communication. Multivariate logistic regression analysis was performed to determine factors associated with loss to follow-up.

**Results:**

The 199 out of 298 screened patients were included in the study. Except for 67 cases with regular follow-up, the rest 132 cases were lost to follow-up. Factors, including age > 60 years (OR = 2.036, *P* = 0.03), monthly income ≤ $583 (OR = 5.568, *P* = 0.0001), education ≤ 6 years (OR = 7.474, *P* = 0.0001), and non-local residence (OR = 5.893, *P* = 0.0001) were associated with the loss to follow-up according to multivariate logistic regression analysis. The most common reasons for loss to follow-up were economic factors and clinic visit distance. The overall case fatality rate (CFR) within 180 days in patients with regular follow-up and patients who stopped anti-fungal treatment during the loss to follow-up was 0% and 19.65%, respectively.

**Conclusion:**

The proportion of loss to follow-up in CPA patients remained high. Age (>60 years), poor financial status, low education, and non-local residence were the key factors associated with the loss to follow-up in this study. Our study reveals the need to optimize the follow-up procedures and improve the patients understanding about the benefits and limitations of follow-up to reduce the CFR.

## Introduction

Chronic pulmonary aspergillosis (CPA) is a chronic progressive infection with a locally invasive presentation caused by *Aspergillus* species (1). The disease is estimated to cause three million cases globally, and patients always experience a poor prognosis with a 5-year survival rate of 17.5–85% (2). It has recently been recognized as a global public health concern (3). With the widespread use of antibiotics, glucocorticoids, immunosuppressants, and the development of organ transplantation and invasive surgical procedures, the incidence rate of infections by special pathogens, especially for pulmonary mycosis, has increased significantly (4). There are three main categories of CPA with overlapping clinical features (5), including chronic cavitary pulmonary aspergillosis (CCPA) with multiple cavities formed and expanded with fungal balls (6), chronic fibrosing pulmonary aspergillosis (CFPA) with multiple solitary lesions leading to substantial pulmonary fibrosis, and subacute invasive pulmonary aspergillosis (SAIA), also known as chronic necrotizing pulmonary aspergillosis (CNPA), which mainly occurs in mild to moderate immune impairment patients having slowly progressive manifestation (7).

Treatment options for CPA according to new consensus guidelines include surgical resection and long-term antifungal therapy. According to the 2017 ERS diagnostic guidelines for pulmonary aspergillosis (8), and 2016 ERS chronic pulmonary aspergillosis diagnosis and treatment management guidelines (9), the recommended initial anti-fungal treatment should last for at least 4–6 months. If the condition worsens during treatment, the treatment plan needs to be revised. If the response to treatment is general, the initial treatment can be extended to 9 months. If the response is good, it is recommended to continue long-term treatment (even lifelong treatment), and to further weigh the pros and cons according to the patient's lung function, drug tolerance, economic status, and other factors. Azole antifungals are commonly used for the treatment of CPA. However, nearly all azole antifungals tend to cause myriads of side effects, including peripheral neuropathy, elevated liver enzymes, heart failure, sun sensitivity, and QT prolongation (10). The side effects may reduce patients' tolerability and lead to premature self-discontinuation, resulting in ultimately poor clinical outcomes (11). Dynamic follow-up after discharge is helpful to enhance patient compliance and reduce mortality (12, 13).

The follow-up work of chronic diseases' medical treatment is often carried out in the form of a telephone interview. Regular follow-up can improve the prognosis and prolong life expectancy (14, 15). Via the follow-up, we can better understand the recent symptoms and nutritional status of patients with CPA, as well as adverse reactions after medication assurance, the occurrence of secondary infections, and disease progression status. All these parameters may help reduce the mortality of CPA. However, the management of patients with CPA after discharge has not been attached with enough importance, especially in China. The high proportion of loss to follow-up deserves our attention.

In this study, we aim to explore the factors associated with loss to follow-up in patients with CPA who previously underwent anti-fungal treatment and assess the impacts on the patients' prognosis after withdrawing anti-fungal treatments during loss to follow-up.

## Materials and methods

### Study design

**Figure 1:**
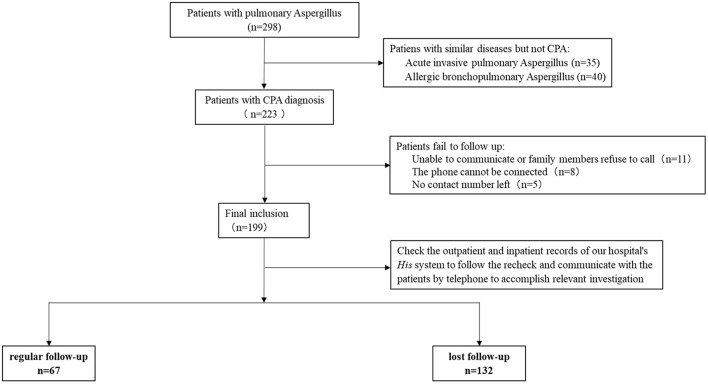
Study flow-chart of this study. A total of 298 patients were initially screened according to the inclusion criteria. After excluding 75 cases of acute IPA and ABPA as well as 24 cases with no telephone contact information or unable to communicate by telephone, 199 patients were finally included in this study.

To explore the factors associated with the high proportion of loss to follow-up in CPA patients and assess the patients' prognosis after withdrawing anti-fungal treatments during loss to follow-up, a prospective, single-center, observational cohort study was designed. Consecutive patients diagnosed with CPA in 2 years were enrolled for this study. Conventional methods, including biopsy, culture, and blood galactomannan test (>80 AU/mL) were performed to detected *Aspergillus*. Pulmonary imaging tests were also performed, and the radiological characteristics were analyzed. Patients were followed up for 1 week ± 1 day, 2 weeks ± 1 day, 1 month ± 4 days, 3 months ± 4 days, and 6 months ± 4 days after discharge. The clinical information and 6-month follow-up results were recorded. As shown in Figure 1, patients were divided into two groups, the regular follow-up group and the loss to follow-up group. Factors, including age (>60 years), financial status (higher than the average monthly income), education (primary school graduation), and distance to hospital (local residence or not) were compared between the two groups.

### Study participants, inclusion and exclusion criteria

In this prospective study, patients with CPA admitted to the Department of Respiratory and Critical Care Medicine, the First Affiliated Hospital of Guangzhou Medical University, Guangzhou, China from March 2017 to November 2019 were enrolled. Patients fulfilling the following inclusion criteria were included in the study: (1) Disease course for at least 3 months; (2) Chest imaging revealed one or more cavities with or without fungal balls or fungal nodules; (3) Microscope and biopsy culture showed direct evidence of *Aspergillus* infection; and (4) Radiological characteristics indicated CPA, including CCPA, CNPA, CFPA, simple aspergilloma, and *Aspergillus* nodules based on relative diagnostic guidelines for pulmonary aspergillosis (8, 16).

The CPA diagnostic criteria were based on the 2017 ESCMID-ECMM-ERS diagnostic guidelines for pulmonary aspergillosis (8), 2016 ERS chronic pulmonary aspergillosis diagnosis and treatment management guidelines (9), and 2016 IDSA: a clinical practice guide for *Aspergillus* infection (17). Briefly, CPA was diagnosed based on evidences of the clinical manifestation, radiological characteristics, length of onset, blood IgG and IgM test, open lung biopsy, and bronchoscopy. Despite open lung biopsy and bronchoscopy, other tests were performed in all enrolled patients.

Patients with single pulmonary cavity containing a fungal ball, with positive serological or microbiological test results indicating *Aspergillus*, and with minor or even no symptoms, and no radiological progression over at least 3 months were diagnosed with simple aspergilloma.

Patients with at least one pulmonary cavity possibly containing one or more aspergillomas or irregular intraluminal material, with positive serological or microbiological test results indicating *Aspergillus*, with significant pulmonary and/or systemic symptoms and overt radiological progression over at least 3 months of observation were diagnosed with CCPA. Severe fibrotic destruction of one lobe with a cavity were simply referred to as CCPA affecting that lobe.

Patients with at least two lung lobes of severe fibrotic destruction complicating CCPA resulting in a major loss of lung function (often accompanied by atelectasis secondary to fibrosis) were diagnosed with CFPA;

Patients with variable radiological features including cavitation, nodules, progressive consolidation with “abscess formation” over 1–3 months, with biopsy and microbiological evidence indicating *Aspergillus* infection were diagnosed with CNPA; It should be noted that acute exacerbation of clinical manifestation and elevation of both *Aspergillus* IgM and IgG could occur in CNPA patients.

Patients with at least one histologically diagnosed *Aspergillus* nodules with no tissue invasion were also enrolled.

The exclusion criteria were as follows: (1) Infections only by pathogens other than *Aspergillus*; (2) Patients diagnosed with acute invasive pulmonary aspergillosis (IPA) and allergic bronchopulmonary aspergillosis (ABPA); (3) Lack of telephone contact information or unable to communicate by telephone; and (4) Clinical data were insufficient. It should be noted that mixed infections with *Aspergillus* were included in this study.

### Data collection and follow-up visits

The clinical information (including sex, age, address, occupation, telephone number, outpatient treatment records, outpatient medication, and expenses) were collected. The patients were followed up within 6 months after discharge, and followed up the lost patients by telephone at the time of 1 month, 3 month, and 6 months after loss to follow-up. All the information was recorded (see Supplementary Table 1). Patients were divided into two groups according to the follow-up results, the regular follow-up group and the loss to follow-up group. Loss to follow-up in this study was defined as patients abandoned regular hospital visits for further examination and buying medicine after discharge. The reasons for the loss to follow-up were summarized. The clinical information and associated factors between the two groups were compared.

The Study protocols were reviewed and approved by the Ethical Committee of the First Affiliated Hospital of Guangzhou Medical University (approval number 2019–26). All the participants were informed about the study and related procedures, and written informed consent of each patient was obtained.

### Statistical analysis

SPSS version 24.0 was used for the statistical analysis. The reasons for the loss to follow-up by telephone were estimated using descriptive analysis. The measurement data conforming to the normal distribution were expressed as mean with standard deviation (x ± SD), and a *t*-test was used for the comparison between the two groups. The measurement data that did not conform to the normal distribution were expressed as median with interquartile range (IQR), and Mann-Whitney-U test was used for the comparison between the two groups. Comparison of counting data was performed using chi-square (χ^2^*)* test or fisher's exact probability test. A rank sum test was used to compare graded data. Multivariate logistic regression analysis was used to analyze the influencing factors of loss to follow-up. *P*-value < 0.05 was considered statistically significant.

## Results

### Participants and epidemiological features of CPA patients

A total of 298 patients were initially screened according to the inclusion criteria. After excluding 75 cases of acute IPA and ABPA as well as 24 cases with no telephone contact information or unable to communicate by telephone, 199 patients were finally included in this study (Figure 1). Among these 199 patients, 140 were male and 59 were female. The average age of all enrolled patients was 58 ± 15 years. There were 92 patients with age ≤ 60 years and 107 patients with age >60 years. The average monthly income of patients was $583. The monthly income of 109 patients was higher than $583, while 90 patients had monthly income lower than this figure. The 105 patients had up to 6 years of elementary school education, and 94 received more education. A total of 55 patients belonged to local residents, and 144 were from remote areas. The details are shown in Table 1.

**Table 1:** Baseline data of patients included in the study and univariate analysis (lost patients vs. regular follow-up patients).

			**Investigation of lost visits**			***χ^2^***	***P-*value**
**Factor**	**Total**		**Lost visit**	**Visiting**	**Lost visit rate (%)**		
Total number of cases	199		132	67	66.8	–	–
Sex	59	Female	42	17	71.19	0.885	0.347
	140	Male	90	50	64.29		
Age	92	[0, 60)	49	43	53.26	13.089	0.0001
	107	[60, ∞)	83	24	0.00		
Monthly income	109	[0, 4,811]	87	22	79.81	19.624	0.0001
	90	(4,811, ∞)	45	45	50		
Local residence	55	Yes	21	34	38.18	26.969	0.0001
	144	No	111	33	77.08		
Length of education	105	[0,6]	92	13	87.62	45.104	0.0001
	94	(6, ∞)	40	54	42.55		
Basic diseases with immune deficiency	61	Yes	45	16	73.77	2.180	0.140
	128	No	87	51	63.04		
Voriconazole monthly expenditure	150	(10,000, ∞)	104	46	69.14	2.458	0.12
	49	[0,10,000]	28	21	57.13		

CCPA was the most common CPA categories of this study, accounting for 62.31% (124/199) of all enrolled patients, followed by CNPA (16.08%, 32/199), aspergilloma (14.57%, 29/199), CFPA (3.52%, 7/199), and aspergillus nodules (3.52%, 7/199). There were 23 patients (13 with CCPA, 9 with CNPA, and 1 with CFPA) died of CPA related illnesses (such as recurrent massive hemoptysis and multiple organ failure) in this study (Supplementary Table 2), the overall 180-day case fatality rate (CFR) was 11.56%.

### Single and multiple factors of loss of follow-up of CPA patients

The 67 patients (including 17 female and 50 male patients) had regular followed-up within 6 months, but 132 cases were lost to follow-up (Figure 1). Of the 132 cases with loss to follow up, the average age was 61.82 ± 12.17 years, and 83 cases were > 60 years old; 45 patients had monthly income higher than $583 (the average monthly income of all enrolled patients); 40 patients had up to 6 years of elementary school education; 21 patients belonged to local residents. The CPA categories in the loss to follow up group patients included CCPA (68.18%, 90/132), CNPA (17.42%, 23/132), simple aspergilloma (8.33%, 11/132), *Aspergillus* nodule (3.03%, 4/132), and CFPA (3.03%, 4/132). There was no significant difference in the severity of CPA during hospitalization (including the length of hospitalization, and hospitalization expenses, etc.) between patients receiving regular follow-up and those lost to follow-up. Voriconazole (96%, 191/199) was the main anti-fungal drug for CPA treatment during the follow-up in both regular follow-up group and loss to follow-up group, followed by itraconazole (2%, 4/199) and posaconazole (2%, 4/199). All deaths were in the loss to follow-up group in this study. The 180-day CFR of CPA in regular follow-up group patients and loss to follow-up group patients was 0% and 17.42%, respectively.

Univariate analysis showed that age > 60 years old, monthly income $583, non-local residence, and length of education ≤ 6 years were suspected influencing factors associated with loss to follow-up of CPA patients (Table 1). Subsequently, logistic regression analysis showed that age, whether local residents or not, monthly income, and education level were independent influencing factors of the loss to follow-up (Table 2). CPA patients older than 60 years were 2.07 times more likely to lose follow-up than those younger than 60 years (OR = 2.036, *P* < 0.05). Non-resident CPA patients were more likely to lose follow-up than locally resident patients (OR = 5.893; *P* < 0.001). Patients with a monthly income of more than $583 had lower possibility of losing follow-up, compared to those with < $583 monthly income (OR = 5.568; *P* < 0.001). The probability of losing follow-up for patients receiving education for more than 6 years was reduced compared to those who have been educated for < 6 years (OR = 7.47; *P* < 0.001).

**Table 2:** Results of multi-factor analysis of the reasons for loss of follow-up.

		**B**	**Standard error**	**Wald**	**Freedom**	***P-*value**	**OR**
Age>60 years old	Yes vs. No	0.711	0.407	3.043	1	0.031	2.036
Monthly income ≤ $583	Yes vs. No	1.717	0.408	17.686	1	0.0001	5.568
Education time ≤ 6 years	Yes vs. No	2.011	0.424	22.496	1	0.0001	7.474
Local residence	Yes vs. No	1.774	0.432	16.848	1	0.0001	5.893

### The cognition to the disease and the reasons of losing follow-up of CPA patients

**Figure 2:**
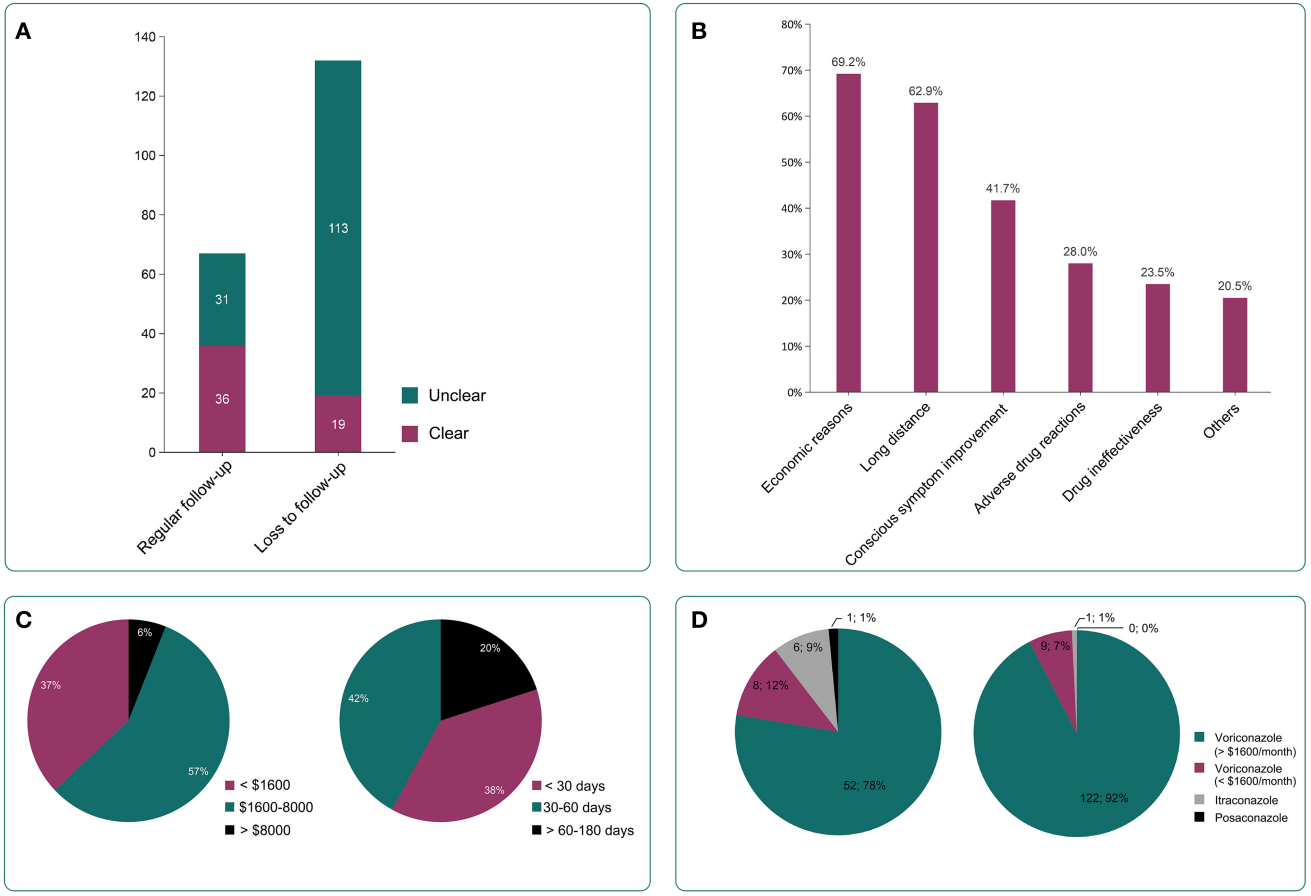
Relevant information of enrolled patients. **(A)** The number of patients with relevant knowledge of CPA (including disease severity, course of treatment, etc.,) in regular follow-up group and loss to follow-up group. Regular follow-up patients had a better understanding of CPA than loss to follow-up group patients (53.8 vs. 14.39%, *P* <0.001). **(B)** The reasons for the loss include economic reasons (69.2%, 90/132), long distance (62.9%, 83/132), conscious symptom improvement (41.7%, 55/132), adverse drug reactions (28%, 37/132), drug ineffectiveness (23.5%, 31/132), and other reasons (including failed appointment for registration, loss of relevant medical records, lack of time, etc.,) (20.5%, 27/132). **(C)** Treatment costs before loss of follow-up (left) and treatment duration before loss of follow-up (right). **(D)** Application of anti-fungal drugs in included patients. Voriconazole (96%, 191/199) was the main anti-fungal drug for CPA treatment during the follow-up in both regular follow-up group and loss to follow-up group, followed by itraconazole and posaconazole (4%, 8/199).

Descriptive analysis about the cognition to the disease between patients with regular follow-up and those lost to follow-up showed that the proportion of patients with relevant knowledge of CPA (including disease severity, course of treatment, etc.) in regular follow-up group was significantly higher than that of patients in loss to follow-up group (53.8 vs. 14.39%, *P* < 0.001). This indicated that the regular follow-up patients have a better understanding of the relevant knowledge of CPA than those lost to follow up (Figure 2A). Telephone communication to the loss to follow-up patients indicated that the main reason for loss to follow-up was economic reasons (69.2%, 90/132), long follow-up distance (62.9%, 83/132), conscious symptom improvement (41.7%, 55/132), and other reasons (including unable to make an appointment for registration, loss of relevant medical records, lack of time, etc.) (20.5%, 27/132). The details are shown in Figure 2B.

### The treatment cost and anti-fungal treatment time of the lost patients before the loss of follow-up

The treatment cost of the lost patient before the loss of follow-up was $2340 ± 2392. The stratification by cost showed that patients spending $1,600–8,000 comprised the largest proportion (57%, 75/132), followed by ≤ $1,600 (37%, 49/132), and > $, (6%, 8/132), as shown in Figure 2C. The median anti-fungal treatment time of these patients before the loss of follow-up was 30 (7–40) days. The anti-fungal treatment time was 30–60 days (42%, 55/132), followed by ≤ 30 days (38%, 51/132) and 60–180 days (20%, 26/132) as shown in Figure 2C. Only 10% (13/132) continue to be treated in the local hospitals after the loss of follow-up to our center.

### Anti-fungal drugs used in two groups of patients

In the two groups of patients, voriconazole (96%, 191/199) was the main anti-fungal drug. A small number of patients were prescribed itraconazole and posaconazole. The monthly cost of the lost patients who used voriconazole was more likely to surpass $1,600. 11% of patients used two or more anti-fungal drugs as shown in Figure 2D. The 23.8% of total patients believed that the drug was ineffective, and 28% of those who lost follow-up thought that the drug had serious adverse reactions.

### Prognosis between the two groups within 6 months after discharge

Despite the comparable severity of CPA during hospitalization, there were no deaths observed in the regular follow-up group patients, while 23 deaths (17.42%) were observed in the loss to follow-up group patients. Further analysis revealed that the most common CPA categories in the deaths was CCPA (56.52%, 13/23), followed by CNPA (39.13%, 9/23) and CFPA (4.35%, 1/23). The mortality rate of patients with CNPA, CFPA, and CCPA was 39.13% (9/23), 25.00% (1/4), and 14.44% (13/90), respectively. There was no death from simple aspergilloma and aspergillus nodules.

## Discussion

In this study, 132 out of 199 CPA patients were lost to follow-up, the follow-up rate was 33.2% (67/199), lower than that of other chronic diseases (18, 19). There was no significant difference in the severity of CPA during hospitalization and the antibiotic regimen after discharge between patients receiving regular follow-up and those lost to follow-up. Age >60 years (OR = 2.036, *P* = 0.03), monthly income ≤ $583 (OR = 5.568, *P* = 0.0001), education ≤ 6 years (OR = 7.474, *P* = 0.0001), and non-local residence (OR = 5.893, *P* = 0.0001) were the main associated factors with the loss to follow-up according to multivariate logistic regression analysis, which confirmed our hypothesis. In addition, compared with patients receiving regular follow-up, loss to follow-up patients had poorer relevant knowledge of CPA and experienced higher 180-day CFR. Our findings indicated age (>60 years), poor financial status, low education, and non-local residence were the key factors associated with the loss to follow-up in CPA patients, and the follow-up procedures needed to be optimized to improve the patients understanding about the benefits and limitations of follow-up, reducing the CFR.

Similar to previous studies, which revealed high medical costs as an important factor influencing the follow-up of patients with chronic fungal infections (such as cryptococcosis) (20, 21), low monthly income was found to be a relevant factor to loss to follow-up in CPA patients in the present study. In addition, low education was another associated factor that should not be ignored. One reason is that patients with a higher level of education and a higher social-economic class are reported to have a high degree of understanding of the disease by grasping the relevant information and hazardness of the disease through the Internet, books, magazines, and other sources (22, 23). The population with low education and low income, on the other hand, pay less attention to the cognition of the disease, thus, less focusing on the follow-up, leading to non-persistent treatment. In addition, regular diagnosis and treatment are easy to stop due to the influence of the surrounding environment. In the present study, 42.3% of patients lost the follow-up due to the improvement of their symptoms, supporting the above view.

Itraconazole and voriconazole are the main drugs for the treatment of chronic pulmonary aspergillosis (9). Most patients with CPA in this study were treated with voriconazole. However, the median time of treatment before the loss of follow-up was only 30 (7–40) days, which was far from the recommended treatment duration (6–9 months) according to relevant guidelines (9). When tracking the lost patients, 85.61% did not know the length of time they needed to use anti-fungal drugs, and did not realize that monitoring the blood concentration is needed while using voriconazole. This situation might lead to the higher 180-day CFR in CPA patients with loss to follow-up than that of other pulmonary fungal diseases (20, 24). The analysis of loss to follow-up and prognosis found that although the proportion of CCPA was higher than that of other types of CPA, the mortality of CNPA after follow-up loss and discontinuation of treatment was much higher than that of CCPA, which was consistent with relevant previously published reports and guidelines (13, 17). CNPA usually causes invasive damage to lung tissue so that progress to acute invasive pulmonary aspergillosis after drug withdrawal in patients lost to follow-up, leading to ultimate worse prognosis.

Voriconazole has great individual differences in pharmacokinetics, and also shows interactions with a variety of drugs. Therefore, it is necessary to carry out therapeutic drug monitoring (TDM) to effectively adjust the drug dose, reduce the potential adverse reactions of drugs, and avoid drug interactions (25). Patients with CPA who were lost to follow-up may have serious adverse reactions due to excessive blood drug concentration or ineffective anti-fungal treatment due to substandard drug concentration. In this study, 23.8% of total patients believed that the drug was ineffective, and 28% of those who lost follow-up thought that the drug had serious adverse reactions. Thus, regular follow-up, especially regular monitoring of TDM, is required for CPA patients to improve the prognosis and reduce the mortality.

This study also has some limitations. This is a single-centered study and the number of patients is relatively small, limiting the generalizability. A total of 24 patients failed to follow up due to lack of telephone contact information or unable to communicate by telephone were excluded, which may cause significant bias. The information based on telephone and other confounding factors raise the risk of reporting bias. The follow-up time after the loss of follow-up was still short, and some other limitations need to be expanded in the next study. Nevertheless, we showed that CPA needed to be included in chronic disease management. The follow-up procedures should be improved (15, 26), and the cognition of patients and their families about the severity of the disease should be strengthen.

## Conclusion

In this study, the factors associated with loss to follow-up in CPA patients were assessed. Age (>60 years), poor financial status, low education, and non-local residence were the key factors associated with the loss to follow-up in CPA patients. The most common reasons for the loss to follow-up were economic factors and clinic visit distance. Patients lost to follow-up may experience worse prognosis with higher 180-day CFR due to premature withdrawal of antifungal treatment. The follow-up procedures needed to be optimized to improve the patients understanding about the benefits and limitations of follow-up for CPA, reducing the CFR.

## Data availability statement

The raw data supporting the conclusions of this article will be made available by the authors, without undue reservation.

## Author contributions

All authors listed have made a substantial, direct, and intellectual contribution to the work and approved it for publication.
