# Supplementary material for: Loss to follow-up associated factors in patients with chronic pulmonary aspergillosis and its impact on the disease prognosis
Source: Front Public Health. 2022 Dec 13;10:1026855. doi: 10.3389/fpubh.2022.1026855 (PMC9792682; doi:10.3389/fpubh.2022.1026855)
Supplement: Supplementary file 1 [file Table_1.DOCX]

**Table S1. Schedule of outpatient return visits for patients with chronic pulmonary aspergillosis (CPA)(translated)**

**Dear Sir/Madam**

**Hello! CPA should be treated in a standardized way for at least 6-9 months. In order to ensure the treatment effect, regular follow-up should be conducted after discharge. During this period, a special medical team will follow up to provide professional advice. According to your actual situation, we have arranged the following time and arrangements for regular return visits.**

| **NAME Gender □Male □Female Age: Hospital ID: Phone No:**  **Address:**  **Education level: □6 years; □9 years;□ 12 years;□16years or more** | | | | | | | | |
| --- | --- | --- | --- | --- | --- | --- | --- | --- |
| **Dignosis and treatment** | **Category of CPA：□ Chronic cavitary pulmonary aspergillosis (CCPA)**  **□ Fugal Ball □ Aspergillus nodules**  **□ Chronic fibrosing pulmonary aspergillosis (CFPA)**  **□Chronic necrotizing pulmonary aspergillosis (CNPA)**  **Diagnosis and treatment plan：**  **□Diagnostic time： □Time of starting treatment：**  **□Treatment plan： □Dosage：**  **□LOSS TO follow up; □LOSS TO contact** | | | | | | | |
| **Time point for further consultation** | **Treatment for 1 week** | **Treatment for 2 week** | **Treatment for 1 month** | **Treatment for 3 month** | **Treatment for 6 month** | **Treatment for 9 month** | **Treatment for 12 month** | **Remarks** |
|  |  |  |  |  |  |  |  |  |
| **Time window** | **±1 days** | **±1 days** | **±4 days** | **±4 days** | **±4 days** | **±4 days** | **±4 days** |  |
| **Review content** | Inquiry,  meidical treatment,  physical examination,  liver function;  Exhalation test;  Blood concentration | Inquiry,  meidical treatment,  physical examination,  Blood concentration  liver function;  Exhalation test;  **Chest CT（Optional）**  **Pulmonary function（Optional）** | Inquiry,  meidical treatment,  physical examination,  liver function;  Blood concentration | Inquiry,  meidical treatment,  physical examination,  liver function;  Exhalation test;  Blood concentration  **Chest CT**  **IgG（Optional）**  **Pulmonary function（Optional）** | Inquiry,  meidical treatment,  physical examination,  liver function;  Exhalation test;  Blood concentration  **Chest CT**  **IgG（Optional）**  **Pulmonary function（Optional）** | Inquiry,  meidical treatment,  physical examination,  liver function;  Exhalation test;  Blood concentration  **Chest CT**  **IgG（Optional）**  **Pulmonary function（Optional）** | Inquiry,  meidical treatment,  physical examination,  liver function;  Exhalation test;  Blood concentration  **Chest CT**  **IgG（Optional）**  **Pulmonary function（Optional）** |  |
| **Treatment plan** |  |  |  |  |  |  |  |  |
| **Hospitalized or not** |  |  |  |  |  |  |  |  |
| **Operation or not** |  |  |  |  |  |  |  |  |
| **Accumulated expenses** |  |  |  |  |  |  |  |  |
| **Note** | **If the treatment is less than 12 month, the medicine can be stopped according to the doctor's evaluation, and the follow-up plan will be adjusted according to the actual situation** | | | | | | | |
