# Supplementary material for: Loss to follow-up associated factors in patients with chronic pulmonary aspergillosis and its impact on the disease prognosis
Source: Front Public Health. 2022 Dec 13;10:1026855. doi: 10.3389/fpubh.2022.1026855 (PMC9792682; doi:10.3389/fpubh.2022.1026855)
Supplement: Supplementary file 2 [file Table_2.DOCX]

**Table S2: Follow up of prognosis of CPA patients within half a year**

|  | **Regular follow-up** | | **Lost patients** | | **Mortality comparison** |
| --- | --- | --- | --- | --- | --- |
| **Types of CPA** | **alive** | **dead** | **alive** | **dead** | **(Regular follow-up vs Lost patients)** |
| **Total number of cases** | 67 | 0 | 109 | 23 | 0% vs 17.42% (*P*<0.01) |
| **Aspergilloma** | 18 | 0 | 11 | 0 | - |
| **Aspergillus nodule** | 3 | 0 | 4 | 0 | - |
| **CCPA** | 34 | 0 | 77 | 13 | 0% vs 14.44% (*P*=0.019) |
| **CNPA** | 9 | 0 | 14 | 9 | 0% vs 39.13% (*P*=0.02) |
| **CFPA** | 3 | 0 | 3 | 1 | 0% vs 25.0% (*P*=0.35) |

Notes: The 23 patients who died were patients who stopped taking drugs after the loss of follow-up. Excluding patients who continued to treat after the loss of follow-up, the mortality was 19.65%. The mortality of CNPA is higher than that of CCPA (39.13% vs 14.44%, *P*<0.05).

Abbreviation: CCPA=chronic cavitary pulmonary aspergillosis, CNPA=chronic necrotizing pulmonary aspergillosis, CFPA=chronic fibrosing pulmonary aspergillosis.
